# Acute Severe Anaphylaxis in Nepali Patients with Neurotoxic Snakebite Envenoming Treated with the VINS Polyvalent Antivenom

**DOI:** 10.1155/2019/2689171

**Published:** 2019-05-02

**Authors:** Sanjib Kumar Sharma, Emilie Alirol, Anup Ghimire, Suman Shrestha, Rupesh Jha, Surya B. Parajuli, Deekshya Shrestha, Surya Jyoti Shrestha, Amir Bista, David Warrell, Ulrich Kuch, Francois Chappuis, Walter Robert John Taylor

**Affiliations:** ^1^B.P. Koirala Institute of Health Sciences, Dharan, Nepal; ^2^Division of Tropical and Humanitarian Medicine, University Hospitals of Geneva, Geneva, Switzerland; ^3^Snake Bite Treatment Centre Nepal Red Cross Society, Chapter Damak, Jhapa, Nepal; ^4^Bharatpur Hospital, Bharatpur, Chitwan 44200, Nepal; ^5^Snake Bite Management Centre Charali, Charali, Jhapa, Nepal; ^6^Nuffield Department of Clinical Medicine, University of Oxford, Oxford, UK; ^7^Institute of Occupational Medicine, Social Medicine and Environmental Medicine, Goethe University, Frankfurt am Main, Germany; ^8^Mahidol Oxford Tropical Medicine Research Unit, Bangkok, Thailand

## Abstract

Diagnosing and treating acute severe and recurrent antivenom-related anaphylaxis (ARA) is challenging and reported experience is limited. Herein, we describe our experience of severe ARA in patients with neurotoxic snakebite envenoming in Nepal. Patients were enrolled in a randomised, double-blind trial of high vs. low dose antivenom, given by intravenous (IV) push, followed by infusion. Training in ARA management emphasised stopping antivenom and giving intramuscular (IM) adrenaline, IV hydrocortisone, and IV chlorphenamine at the first sign/s of ARA. Later, IV adrenaline infusion (IVAI) was introduced for patients with antecedent ARA requiring additional antivenom infusions. Preantivenom subcutaneous adrenaline (SCAd) was introduced in the second study year (2012). Of 155 envenomed patients who received ≥ 1 antivenom dose, 13 (8.4%), three children (aged 5−11 years) and 10 adults (18−52 years), developed clinical features consistent with severe ARA, including six with overlapping signs of severe envenoming. Four and nine patients received low and high dose antivenom, respectively, and six had received SCAd. Principal signs of severe ARA were dyspnoea alone (n=5 patients), dyspnoea with wheezing (n=3), hypotension (n=3), shock (n=3), restlessness (n=3), respiratory/cardiorespiratory arrest (n=7), and early (n=1) and late laryngeal oedema (n=1); rash was associated with severe ARA in 10 patients. Four patients were given IVAI. Of the 8 (5.1%) deaths, three occurred in transit to hospital. Severe ARA was common and recurrent and had overlapping signs with severe neurotoxic envenoming. Optimising the management of ARA at different healthy system levels needs more research. This trial is registered with NCT01284855.

## 1. Introduction

Snake antivenoms are the only specific treatments for snakebite envenoming; they save lives but are associated with acute pyrogenic reactions, due to endotoxin contamination whilst in production, and acute anaphylaxis [1].

The mechanisms underlying antivenom-related anaphylaxis (ARA) are uncertain and are probably a combination of complement activation, a type I hypersensitivity reaction, non-allergen-specific activation of mast cells triggered by the antivenom impurities and immune priming due to the venom itself [2, 3]; indeed, anaphylaxis has been reported in snake handlers after >1 envenoming [4]. The reported rates of ARA range from <5 up to <90% depending on the quality of the antivenom used and the diligence in recording events [5–12]. One report from Thailand suggested higher rates in cobra-bitten (~12%) vs. viper-bitten (~2%) patients [13] whereas a larger Indian study found the opposite relationship [14].

ARA has the same clinical features as other causes of anaphylaxis. Common (20-50%) features include urticaria, tachycardia, hypotension, tachypnoea, dyspnoea, wheeze, and angioedema. Restlessness, agitation, confusion, laryngeal obstruction, stridor, sinus bradycardia, and relative bradycardia with hypotension are seen less frequently [8, 9, 15, 16].

Severe life-threatening ARA characterised by shock, hypoxia, and reduced consciousness/confusion were low (<2 – ~7%) in studies from Ecuador [17], Papua New Guinea [8], Australia [15], and India [18] but were ~22–33% in Sri Lanka [6, 10, 19], Bangladesh [11], Pakistan [20], and Laos [21]. ARA is reduced substantially by administering subcutaneous adrenaline (SCAd) before antivenom is given [6].

Intramuscular adrenaline (IMAd) is the treatment of choice for acute ARA, but ARA may respond poorly (protracted anaphylaxis) or recur later (biphasic anaphylaxis) [22]. Giving additional doses of antivenom to treat progressive or recurrent envenoming is another important cause of recurrent ARA [9]. The optimal management of protracted or recurrent ARA is unclear. One recommendation is an intravenous adrenaline infusion (IVAI) [16] with tight control of the blood pressure to avoid adrenaline-induced toxicity like hypertension and intracranial haemorrhage [23].

Most venomous snake bites in Nepal are caused by spectacled and monocled cobras,* Naja Naja *and* Naja kaouthia*, and the common krait,* Bungarus caeruleus* [24]. Their neurotoxic venoms cause death by paralysing the bulbar and respiratory muscles. Indian-manufactured, equine-derived, polyvalent antivenom raised against the venoms of* B. caeruleus*,* Daboia russelii* (Russell's viper),* Echis carinatus* (saw-scaled viper), and* N. naja* is the only antivenom available in Nepal and, in one study, was associated with severe ARA in ~22% of recipients [19].

We reported previously the efficacy and tolerability of high vs. low dose antivenom in neurotoxic envenomed patients [25]. Herein, we focus on severe ARA.

## 2. Methods

### 2.1. Study Design and Site

Study details are published elsewhere [25]. Briefly, this double-blind, placebo-controlled trial took place from April 2011 to March 2013 at Bharatpur Hospital, a tertiary referral hospital with an intensive care unit (ICU), Bharatpur, and two snakebite treatment centres: (i) Snake Bite Treatment Centre, Nepal Red Cross Society, Damak, and (ii) Snake Bite Management Centre, Charali, which are 60 km (1.5 h) and 100 km (2.5 h), respectively, from the B.P. Koirala Institute of Health Sciences (BPKIHS), a university hospital with an ICU. Transferred patients to BPKIHS were accompanied by a doctor. Data analysis in this short report was descriptive.

Ethical clearance was obtained from the B.P. Koirala Institute of Health Sciences Ethics Committee (approval n°ACA-575-/067/068), the Nepal Health Research Council (approval n°986), and the Geneva University Hospitals Ethics Committee (approval n°08-192). All study participants gave written informed consent to participate in this study.

### 2.2. Inclusion/Exclusion Criteria

Recruited patients were aged ≥ 5 years (y), with signs of neurotoxic envenoming, assessed using a neurotoxicity score (NS, Box 1), who/whose guardians gave signed, informed consent. Excluding criteria were (i) pregnant or breast feeding women, (ii) presentation > 24 h, (iii) patients needing immediate mechanical ventilation [respiratory distress, no gag reflex, paradoxical breathing and/or oxygen saturation (SpO_2_)] < 90% on room air], (iv) known allergy to horse proteins, (v) patients (a) with an underlying neuromuscular disease, (b) with a proven viper bite, and (c) who had received antivenom earlier at another health centre.

### 2.3. Antivenom Dose and Adjunct Treatments

We used the VINS manufactured polyvalent antivenom (VINS Bio-pharmaceuticals Corp. Ltd., Mumbai, India); all antivenom came from one batch: #01AS11004. This antivenom contains horse derived F(ab')_2_ antibody fragments against* B. caeruleus*,* D. russelii*,* E. carinatus,* and* N. naja *from India and is associated with severe ARA rates ranging from 4 [26] − 21% [19].

Patients were randomised to either the Nepali recommended low dose (LD) regimen: 6 vials, 2 by IV push over 5-10 m, then 4 infused over 4 h (1h & 3h infusions), or high dose (HD) antivenom: 10 vials by IV push over 5-10 m, then 8 vials infused over 1 h and 3 h normal saline infusion to maintain the blind. Persisting neurotoxic signs were treated with 4h infusions of 4 vials (LD arm) or saline (HD arm). Acute neurological deterioration (i.e., increased NS) was treated with 2 vials (LD arm) or 5 vials (HD arm) by IV push.

All patients received neostigmine to enhance neuromuscular transmission [27] and atropine to prevent the muscarinic effects of neostigmine (hypersalivation, colic, pulmonary oedema, and sinus bradycardia).

### 2.4. Patient Monitoring

Patients were monitored hourly until the NS became 0 (i.e., complete resolution of neurotoxic signs) then 6 hourly and included emergent symptoms and signs, vital signs, NS, and SpO_2_ measured by finger oximeter [MD300D (adults), MD 300C5 (children), Vandagraph, United Kingdom]. Patients were followed up on days 7 and 21 and at 6 months.

### 2.5. Reporting of Severe Anaphylaxis

Severe acute anaphylaxis meets the definition of a serious adverse event (SAE), i.e., life-threatening, prolongs inpatient stay, or results in death [28]. All clinical details were recorded on SAE forms by the site investigator under the supervision of the study site principal investigator (SKS) who was on call 24 h/day. All SAE reports were reviewed by EA, FC, & WRJT and then sent to the ethics committees of the BPKIHS and Geneva University Hospitals, the Nepal Health Research Council (NHRC) and the Drug Safety and Monitoring Board (DSMB) for comments.

For this report, the senior author reviewed again all SAE forms and associated communications to reconstruct the clinical picture from patient admission to discharge or death. Clinical events, defined as important symptoms or signs, were identified and the following details were recorded: (i) their timing, (ii) how often they occurred, (iii) what their causes were, (iv) how and at what times they were treated, and (v) outcomes. Data were entered into Microsoft Excel and analysed [descriptive analyses and Mann–Whitney U test (continuous data)] in Stata v14 (Stata Corporation, USA).

### 2.6. Prevention of Anaphylaxis

Premedication with SCAd was given in the second snakebite season in 2012 following the report by de Silva et al. [6]. The dose was 0.25 mL (patients aged ≥ 13 y), 0.2 mL (11-12 y) and 0.125 mL (5-10 y) of a 1:1,000 solution of adrenaline.

### 2.7. Definition and Treatment of Anaphylaxis

We used the definition recommended by the National Institute of Allergy and Infectious Disease and Food Allergy and Anaphylaxis Network, “anaphylaxis is a serious allergic reaction that is rapid in onset and may cause death” [29].

All clinical teams received prestudy and refresher trainings in the recognition and treatment of anaphylaxis, including (i) a list of key symptoms and signs (itching, urticaria, swollen lips or tongue, angioedema, dry cough, wheezing, stridor, hoarse voice, ‘lump in throat', nausea, vomiting, abdominal colic, diarrhoea, hypotension, and shock), (ii) ABC of resuscitation (airway: obstruction/compromise, breathing: tachypnoea, wheezing, and circulation: hypotension or shock +/- poor peripheral circulation), (iii) intubation and the use of an Ambu bag. We stressed the urgency of treating anaphylaxis when the first sign/s consistent with ARA appeared, irrespective of their severity [30], which consisted of IMAd, IV hydrocortisone, and IV chlorphenamine, following international guidelines [30–32]. Training did not include performing a cricothyrotomy or a tracheotomy.

For patients who had experienced ARA but needed more antivenom to treat envenoming, antivenom infusions were resumed when patients were either haemodynamically stable or when the treating physician thought the anaphylaxis was clinically resolved. A reappearance of ARA was treated as above, but we later replaced this practice with IVAIs to “cover” additional doses of antivenom infusion, following the regimen of Brown et al. [16]. IVAIs were adopted because (i) it was sometimes difficult to decide when ARA had resolved fully and so whether it was “safe” to restart the antivenom infusion and (ii) to prevent multiple injections of IMAd if physicians thought patients were having ongoing ARA during the antivenom infusions. Antivenom pushes (as above) for acute neurological deterioration were not covered by adrenaline.

## 3. Results

### 3.1. Demographic and ARA Summary Data

**Figure 1:**
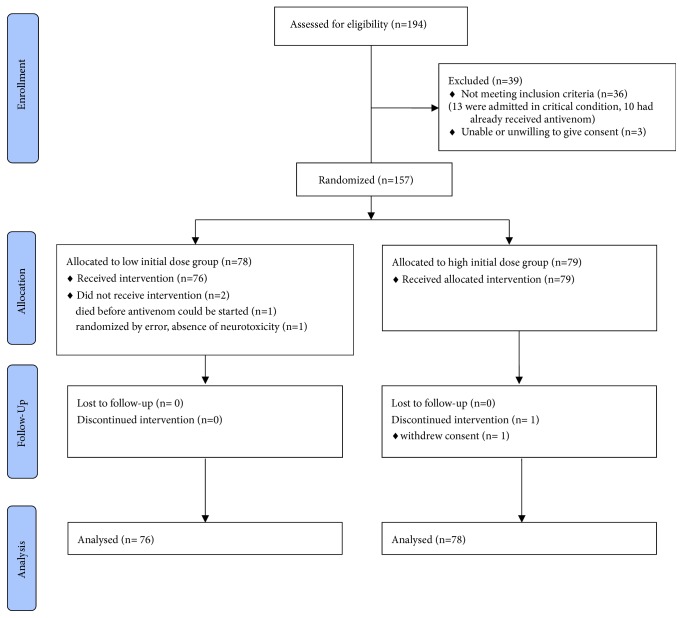
Trial profile.

155 patients with neurotoxic envenoming received at least one dose of antivenom and are included in this analysis, including one patient who later withdrew from the study (Figure 1).

In total, 13 [8.4%, 95% confidence interval (CI) 4.9-13.8%] patients had severe ARA, three were children aged 5, 6, and 11 y and 10 adults, aged 18–52 y (Table 1). Snake identification, by an expert herpetologist or polymerase chain reaction of bite swabs, was possible in six patients.

**Figure 2:**
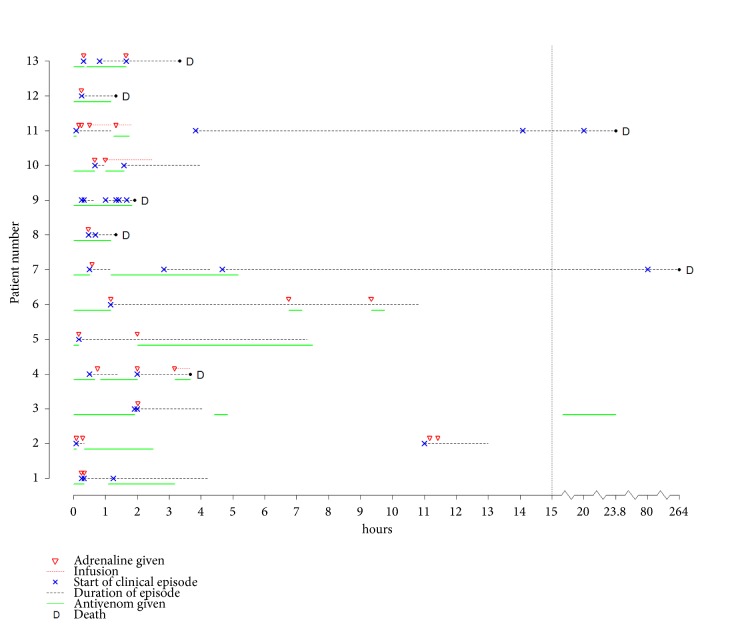
Time course of antivenom administrations, clinical events, and adrenaline treatment. Time 0 is the start of antivenom administration.

Over time, these 13 patients had 64 clinical events: two patients had 3 events, six had 4 events, two had 5 events, and three had 8 events (Table 2). Some events led rapidly to a cascade of additional events whilst others were separated by large time periods (Figure 2).

Median (range) times to the first clinical symptom or sign consistent with ARA after the most recent dose of antivenom were (i) 19 (5-115) minutes (m) after the first IV push of antivenom, (ii) 52.5 (11-155) m (second dose), and (iii) 75 (30-770) m (third dose). SCAd was given to 6 patients in 2012 and four and nine patients received LD and HD antivenom, respectively, but the times to the first ARA event were not significantly different between (i) SCAd recipients 17 (5-40) m vs. nonrecipients 30 [5-115] m (p=0.5) and (ii) LD 14.5 (5-40) m vs. HD 28 (5-115) m regimens (p=0.4).

There were 8 deaths (#s 6–13, Table 3), for a case fatality rate of 5.16 (2.25 – 9.9)%. Five occurred in Bharatpur Hospital. The median time to death was 3.5 h [IQR 1.6–18.4 h (range 1.3 h–11 d). None of the patients who died had acute features of a cholinergic crisis.

### 3.2. ARA Clinical Features & Management

The clinical features were similar between the LD and HD patients (Table 2). Rash, documented as urticaria, erythema, or ‘itchy rashes' were the most common ARA manifestation and occurred during the first ARA episode in 10 patients (Tables 2, 3, and 4). Five patients had other concomitant signs of ARA whilst the other five all went on to develop other features of ARA over time. Severe ARA signs included dyspnoea alone or with wheezing, hypotension, shock, restlessness, and respiratory or cardiorespiratory arrest; two patients with dyspnoea manifested as gasping respirations (#8, #12). Early (#1) and late (#11) laryngeal oedema (E & LLO) were seen in two patients. The ELO occurred 35m into the resumption of his first antivenom infusion (interrupted because of an itchy rash), corresponding to 95 m after the IV push; clinical signs were noisy breathing, cough, and a fall in SpO_2_ to 63%. The patient with LLO developed a hoarse voice 8 h 35 m after his antivenom infusion had finished (11h after IV push). Stridor was not a feature of either E or LLO.

In most ARA-diagnosed patients, antivenom was stopped and IMAd given almost immediately after the ARAs were recognised (Figure 1), followed by IV hydrocortisone and IV chlorphenamine. Nebulisers were given to one patient (#8) without IMAd who continued to receive antivenom without deterioration. Two patients (#s5 & 10) received IMAd after IV hydrocortisone at 6 and 15 m after the start of their first ARAs without deleterious effects. One and three patients were given IV adrenaline and SCAd, respectively, instead of IMAd. Four patients (#s1, 3, 10, 12) were admitted to Bharatpur Hospital and treated with five adrenaline infusions so antivenom could be restarted following antecedent reactions: (i) patient 1: initial antivenom induced rash had resolved, (ii) patient 3: it was unclear if the initial episode of severe ARA had resolved; the patient was ventilated and needed additional antivenom, (iii) patient 10: the patient had partial resolution of antivenom induced rash, and (iv) patient 12: despite two injections of IMAd, patient's rash remained unresolved; IVAI was used to complete the initial IV push of antivenom.

### 3.3. Deaths in Patients with Indeterminate Clinical Features

Six patients (#s 6, 7, 8, 9, 12, 13) had events with clinical features consistent with severe ARA and severe neurotoxic envenoming, although they were all considered to be antivenom-related at the time by the treating physicians (Table 3). Patient 7, whose presentation included dyspnoea, suffered a respiratory arrest and sinus bradycardia 15 m after starting antivenom; despite IMAd, intubation and oxygen, she had a cardiac arrest and died in the ambulance on the way to hospital. The other five patients had increasing neurotoxic scores (n=4), sudden cardiac arrest (n=1), dyspnoea without wheeze (n=1), gasping respiration (n=1), falling oxygen saturation (n=4), restlessness (n=2), hypotension (n=1), and sinus bradycardia (n=4, 5 episodes) that was followed rapidly by death. At the time of these clinical events, patient 9 was on an antivenom infusion, three had received IV push injections for neurological deterioration, and one was under observation in the ICU.

### 3.4. Other Deaths

One patient died of LLO (#11) and the other of anaphylactic shock (#10). Both patients died in the ambulance during hospital transfer.

## 4. Discussion

Our study has documented a relatively high rate (~8%) of VINS antivenom associated ARA that was clinically clear-cut in seven patients but clouded in six mostly by a mix of dyspnoea, restlessness, and increasing neurological scores, all consistent with envenoming.

Acute anaphylaxis is a predictable toxicity of antivenom. The Indian-manufactured antivenoms, used commonly in south Asian countries, are associated with rates of severe ARA as high as ~20 to 40% [3, 6, 10, 19]. Recurrent ARA may recur acutely when additional doses of antivenom are given as infusions or IV pushes and “unexpectedly” as biphasic anaphylaxis hours after the apparent resolution of antecedent episode of ARA [9, 21]. Moreover, patients may “unexpectedly” deteriorate clinically because of a recrudescence of their envenoming despite an initially good clinical response to antivenom. We faced such challenges in several patients.

The most difficult patients were those who developed restlessness and/or acute dyspnoea/gasping respiration with (e.g., #s13, 8) or without (e.g., #12, 9) an increase in the NS score and patients who deteriorated rapidly, culminating in a cardiac/respiratory arrest, whether they were treated for ARA (#6) or not (#7). Sinus bradycardia was an ominous sign that preceded cardiac arrest and death. With an acute increase in NS, clinicians may believe the acute clinical deterioration is exclusively due to worsening envenoming. However, they should consider whether an earlier antivenom infusion or IV push is contributing either acutely or as biphasic ARA and should have a low threshold for treating with IMAd.

To increase clinical awareness, we suggest several “danger” signs that require urgent assessment such as sinus bradycardia, relative bradycardia and hypotension [33], dyspnoea alone or with wheezing, hoarse voice, restlessness, and nonspecific acute clinical deterioration. These signs may occur in isolation or within a picture of worsening neurotoxicity that may be confusing and lead clinicians not to consider ARA.

Five patients developed dyspnoea without wheezing; two (one as gasping respiration) did not have a concomitant decline in NS whilst one gasping patient did. Although wheeze is a classic sign of acute anaphylaxis, dyspnoea alone is well documented and was more common than wheeze in two large clinical series. In one ARA study, ~16% (32/198) patients developed hypoxia, 3% wheeze and hypoxia and 3.5% wheeze alone [10] and, in a study of all cause anaphylaxis (n=1,149), dyspnoea alone was present in 29% patients vs. 13% who had wheeze [34]. Acute dyspnoea without wheezing is also a well-characterised feature of progressive neurotoxic envenoming (leading to insufficient respiratory muscle strength and increasing NS) which requires rapid treatment with antivenom +/- assisted ventilation. Moreover, dyspnoea should always prompt the exclusion of mechanical blockage of the airway (e.g., a prolapsed tongue), and faulty intubation (e.g., into one bronchus) in ventilated patients.

Two patients developed laryngeal oedema neither of whom had stridor. The LLO patient had recovered from his earlier mild ARA and envenoming and his hoarse voice developed 8.5 h postantivenom infusion. It is unclear whether LO developed late and progressed rapidly (i.e., biphasic anaphylaxis) or insidiously to become clinically manifest only when the oedema was severe. He was unresponsive to adrenaline and hydrocortisone and the extent of the LO precluded endotracheal intubation. Without surgical equipment and skilled staff to relieve his upper airway obstruction (UAO), he died. The risk of laryngeal obstruction/oedema/stridor/throat tightness was 2% (1/48) in snakebite victims in Australia [15].

UAO is classed as both moderately severe [34] and life-threatening [31]. In settings where intubation is problematic, mechanical ventilators are several hours away, and death during hospital transfer is well described [35], UAO is better classed as life-threatening. In retrospect, had we anticipated that LO might prevent intubation, we would have added training on cricothyrotomy/tracheotomy together with the necessary equipment.

During the study, our guidelines on treating recurrent ARA changed from treating each episode with IMAd to IVI to cover subsequent antivenom infusions [16]. Four patients, all enrolled at Bharatpur Hospital, received adrenaline infusions. Given the potential dangers of IVAI (e.g., cardiac arrhythmias, acute hypertension, and haemorrhagic stroke), the need for close monitoring, e.g., 3−5 m [36] and infusion titration, IVAIs are suitable only where there are well-trained ICU or emergency staff. More research is needed to optimise the management of patients with antecedent ARA who require repeated antivenom infusions or IV antivenom pushes for acute neurological deterioration.

We had a crude fatality rate (CFR) of ~5%, higher than the 1.3% reported by de Silva et al. in their large (n=1007) trial that was conducted in hospitals with experience of snakebite management [6] but lower than the 13 [37] to 20%, with marked interclinic variation [38], reported previously in Nepal. Nevertheless, these data support a relationship of limited resources and skills and higher CFRs.

To conclude, we have highlighted the risks of severe ARA in neurotoxic envenomed patients and challenges in managing patients where resources are limited. Clinicians should have a low threshold for treating of ARA when the clinical picture is not clear-cut and ARA cannot be excluded. More research is needed to define optimal treatment strategies in different health settings, especially snakebite centres.

## Figures and Tables

**Box 1:**
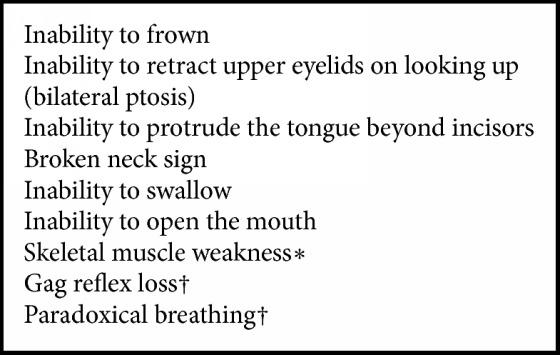
Signs of neurotoxicity. One point is given for each feature present to calculate the neurotoxicity score. *∗*: defined as < 3 on the MRC scale, †: clinical indications for mechanical ventilation.

**Table 1:** Baseline characteristics of the 13 patients by dose of antivenom.

	High dose antivenom	Standard dose antivenom
	n=9	n=4
Age	23 (5–52)	34.5 (6–52)
Sex female:male	2:7	1:3
Received subcutaneous adrenaline	4	2
Snake species		
*Naja naja*	0	1
*Bungarus caeruleus*	3	1
*Bungarus niger*	1	0
Unidentified	5	2
Neurological score	2 (1–4)	3 (2–4)

**Table 2:** Details of the clinical events in 13 neurotoxically envenomed patients with postantivenom serious adverse events.

Patient #	NS D0	AVR	CR arrest	Shock	Hypotension	SB	Dyspnoea	Wheeze	Cyanosis	Cough	↑ NS	Frothy secretions	P Resp	LO	VAP	Restless	Drowsy	Vomiting	Rash	Soft tissue oedema	Fever	Total	Outcome
13	2	H	1	-	-	-	-	-	-	-	2	1	-	-	1	1	-	1	1	-	-	8	D
12	2	H	2	-	-	2	1	-	-	-	-	-	-	-		1	1	-	1	-	-	8	D
11	2	L	-	-	-	-	1	-	-	-	-	-	-	1	-	-	-	-	1	1*∗*	-	4	D
10	2	H	1	1	-	-	-	-	-	-	-	-	-	-	-	-	-	-	1	1†	1	5	D
9	2	L	1	1	-	-	1	-	-	-	-	-	-	-	-	-	-	-	1	-	-	4	D
8	2	H	1	-	-	1	1	1	-	-	1	1	-	-	-	-	-	1	1	-	-	8	D
7	3	H	2	-	-	1	1		-	-		-	-	-	-	-	-	-	-	-	-	4	D
6	3	H	1	-	1	1	-	-	-	-	1	-	1	-	-	-	-	-	-	-	-	5	D
5	2	H	-	-	1	-	1	1	-	-	-	-	-	-	-	-	-	-	1	-	-	4	R
4	4	H	-	-	-	-	1	1	1	-	-	-	-	-	-	1	-	-	-	-	-	4	R
3	4	L	-	-	1	-	1	-	-	-	-	-	-	-	-	-	-	-	1	-	-	3	R
2	1	H	-	1	-	-	-	-	-	-	-	-	-	-	-	-	-	1	1	-	1	4	R
1	4	L	-	-	-	-	-	-	-	1	-	-	-	1	-	-	-	-	1	-	-	3	R

Total			9	3	3	5	8	3	1	1	4	2	1	2	1	3	1	3	10	2	2	64	

NS D0: neurotoxicity score on Day 0 (baseline); AVR: antivenom regimen; H: high-dose antivenom regimen; L: low-dose antivenom regimen; CR: cardiorespiratory; SB: sinus bradycardia.

P Resp: paradoxical respiration; LO: laryngeal oedema; VAP: ventilator associated pneumonia; D: died; R: resolved.

*∗*: unilateral lower eyelid swelling.

†: recorded as angioedema, exact anatomical location unknown.

**Table 3:** Clinical notes on the eight patients with neurotoxic envenoming who died.

Patient #	SCAD	Antivenom dose	Clinical description	Time to death in h*∗*	Commentary
Gender/					
Age					
#13 Male 25 y	N	H	30m after IV push developed generalised erythematous rash. Treated with SC adrenaline x 2 & IV hydrocortisone. Antivenom infusion restarted when rash resolved. ~1.5h later became restless & NS increased from 2 to 4. Treated with IV AV push but his NS remained stable at 4 (1h post push). Another hour later (i.e. 2h after IV push), he had a sudden cardiorespiratory arrest. Intubated, resuscitated successfully, was stable but drowsy and continued on mechanical ventilation. Frothy secretions in ET tube treated with atropine. NS became 0 but he was unable to be extubated. Developed ventilator associated pneumonia and treated with antibiotics. Laryngeal spasm occurred during tracheostomy resulting in death.	264	Had an initially mild ARA. His later restlessness is consistent more with worsening envenoming (increase in NS) than delayed recurrent ARA but the sudden CR arrest is consistent with delayed ARA due to the earlier IV antivenom pushes. Died of complications of snake bite, laryngeal spasm during tracheostomy.

#12 Male 11 y	Y	H	Generalised itching & urticaria developed 5m after IV push started. Treated correctly. IV adrenaline infusion started to cover rest of IV push & antivenom infusion when ARA had resolved. Later at T0+3.8h (2h after antivenom infusion stopped), patient became drowsy & restless with neurotoxicity score=0. Not treated for ARA. Sent to intensive care unit for monitoring. 10h later found gasping. Intubated & improved on oxygen. 6h later fall in SpO_2_, sinus bradycardia, asystole, DC shocked and reverted to sinus tachycardia. Stable but 3.5h later another episode of sinus bradycardia and asystole. Resuscitation unsuccessful.	23.8	Initial itching and urticaria are typical features of mild ARA. Cause of later clinical picture was unclear but is consistent with delayed recurrent anaphylaxis.

#11 Male 6 y	N	L	Urticaria and unilateral eye oedema developed 5m after IV push started. Treated with x 2 SC adrenaline. ARA resolved. Antivenom restarted and stopped when neurotoxic signs disappeared. Patient later developed a hoarse voice (11h from T0, 8.5h since antivenom stopped) that worsened despite treatment with IM adrenaline and IV chlorphenamine. Dyspnoea and falling SpO_2_. Intubation attempt failed because laryngeal oedema was severe. Patient was transferred but died in the ambulance.	13	Late laryngeal oedema is consistent with delayed recurrent ARA.

#10 Male 19 y	N	H	First indication of anaphylaxis was itching during antivenom infusion (30m after IV push). Antivenom stopped. Treated with chlorphenamine but IM adrenaline given 15m later when rash appeared. Antivenom restarted as rash was resolving. 1h 10m later while on antivenom infusion, patient became shocked with falling SpO_2_ and development of angioedema. Resuscitated, intubated & transferred but died in the ambulance.	3.7	Clinical picture of ARA.

#9 Male 18 y	Y	L	Anaphylaxis manifested as mild urticaria 19m after IV push, treated with IM adrenaline & resolved. AV infusion restarted. Developed dyspnoea without wheezing & without an increase in NS (static at 2). Treated with oxygen but SpO_2_ fell to 70%. Then AV stopped and treated appropriately for ARA but progressed rapidly to cardiorespiratory arrest & died despite resuscitation.	3.3	Decline in respiratory function without wheezing was thought initially to be envenoming related. Poor response to ARA treatment after fall in SpO_2_ which was probably ARA related.

#8 Male 33 y	Y	H	Developed vomiting 15m after IV push followed by urticaria and dyspnoea with wheezing 5m later. Treated with salbutamol and ipratropium inhalations. No IM adrenaline given. Antivenom continued (NS=2). 40m later developed increased NS of 4 (IV AV push given) that, 20m later, increased to 6 associated with frothy secretions and muscle weakness (IV AV push given again). Became restless with gasping respirations, BP 90/60 & SpO_2_ 80%, pulse fell from 140 to 55/m (sinus bradycardia, given IV atropine). Intubated, manually ventilated, then cardiac arrest and died despite resuscitation.	1.9	Clinical picture dominated by rapidly progressive envenoming despite treatment with antivenom pushes. Patient did not receive IM adrenaline for initial episode of mild anaphylaxis nor adrenaline cover for the IV pushes, nor IMAd for possible ARA.

#7 Female 5 y	Y	H	Presented with abdominal pain, vomiting, ptosis, tachypnoea (RR 40/m), tachycardia (120/m) and central cyanosis (SpO_2_ 60%) treated with oxygen (SpO_2_ rose to 90%) before antivenom. 15m after AV push & while on AVI had a respiratory arrest associated with sinus bradycardia (50/m). Immediate intubation was followed by a cardiac arrest. Resuscitated with IV adrenaline & 300 mL IV fluid bolus; AVI continued. Pulse detected by oximeter but no recordable blood pressure. Decision made to transfer to hospital. Second cardiac arrest (exact time not noted) followed by death despite resuscitation in ambulance.	1.3	Clinical picture dominated by poor respiratory status before antivenom associated with NS score of 3. Respiratory arrest after antivenom followed by cardiac arrest. Given the rapidity of the events, ARA may have contributed to the clinical picture.

#6 Male 51 y	Y	H	Rapid deterioration in cardiorespiratory function associated with sinus bradycardia and increasing NS. Culminated in a cardiorespiratory arrest and failed resuscitation. Treated for anaphylaxis and given IV antivenom push.	1.3	Treated for worsening envenoming and ARA. Clinical picture dominated by apparent worsening of envenoming that may have masked features of anaphylaxis. Died despite treatment for ARA

*∗*: time from the start of the intravenous push (T0) to the time death was certified. SCAd, subcutaneous adrenaline, IMAd: intramuscular adrenaline, SC: subcutaneous, IV: intravenous, IM: intramuscular.

h: hour, m: minute, y: years, NS: neurotoxicity score, AV: antivenom, AVI: antivenom infusion, ET: endotracheal tube, SpO_2_: oxygen saturation, ARA: antivenom related anaphylaxis.

**Table 4:** Clinical notes on the five patients with neurotoxic envenoming who survived.

Patient #	SCAD	Antivenom dose	Clinical description	Commentary
Gender/				
Age				
#5 Female 23 y	N	H	1.55h after IV push & during AV infusion, developed acute wheezing & rash on forehead. AV stopped. Treated with nebulised salbutamol. 5m later, wheeze became worse, P-144/m, BP fell to 90/50, SaO_2_ 90%. AV stopped immediately. Treated with IV hydrocortisone, chlorphenamine & N saline, followed by IMAd (6m delay). Transferred to ICU for observation. ARA resolved fully after 95m. AV restarted with no further ARAs.	Developed classic features of ARA. Additional doses of AV did not result in additional ARAs despite no prophylactic SCAd or IVAI.

#4 Male 20 y	N	H	1.5h after IV push & during AV infusion, developed acute restlessness, wheezing & cyanosis. Respiratory rate 32/m, SpO2 50%, P-76 BP160/100. AV stopped immediately. Treated with IMAd, IV hydrocortisone, oxygen, then intubated in ICU. Needed two boluses of AV in the ICU; both covered with SCAd. No additional ARAs & made a full recovery.	Developed classic features of life threatening ARA with rapid decline in respiratory function necessitating intubation.

#3 Female 53 y	N	L	10m after start of IV AV push, developed itchy red rash on upper arms, chest abdomen with respiratory distress. Tachycardia and fall in blood pressure (no measurements recorded). AV stopped immediately. Treated with 0.5 mg IVAd x 2, IV hydrocortisone & chlorphenamine & intubation. Rash resolved completely. IVAV restarted 15m after rash resolved under cover of IVAI started to cover. No additional ARAs. Extubated & made full recovery.	Developed classic features of life threatening ARA with rapid decline in respiratory function necessitating intubation. IV rather than IMAd given to treat ARA.

#2 Male 52 y	N	H	15m after start of IV AV push, developed urticaria. Treated with SCAd but AV not stopped. 5m after rash, patient became shocked with an unrecordable BP and cool peripheries. AV stopped. Treated with 1 mg IVAd, hydrocortisone, saline bolus, atropine, second dose of neostigmine & atropine. Stabilised & after 10m signs were P-99/m, BP 90/50, SpO_2_ 90%. AV infusion restarted followed 10m later by a pyrogenic reaction (fever & chills). Treated symptomatically & with 3^rd^ dose of neostigmine & atropine, AV stopped temporarily then continued until resolution of envenoming. No additional ARAs noted.	Initial ARA was a red rash that was treated with SCAd rather than IMAd. AV not stopped and may have resulted in life threatening ARA. Recommencement of AV not covered by adrenaline but no additional ARAs. Pyrogenic reaction was short lived.

#1 Male 51 y	Y	L	40m after IV push & during AV infusion, developed red rash. AV stopped & treated with 0.5 mg IVAd, IV hydrocortisone & chlorphenamine. Rash resolved after 20m & AV infusion restarted under IVAI. 35m later developed cough, noisy breathing & fall in SpO_2_ to 63%. Acute laryngeal oedema suspected and transferred to ICU for intubation.	ARA started with a red rash and resolved with treatment. AV infusion restarted with IVAI but it did not prevent laryngeal oedema.

SCAd: subcutaneous adrenaline, IMAd: intramuscular adrenaline, SC: subcutaneous, IV: intravenous, IM: intramuscular, h: hour, m: minute, y: years, NS: neurotoxicity score.

AV: antivenom, SpO_2_: oxygen saturation, ARA: antivenom related anaphylaxis.

## Data Availability

The data that are presented in this anaphylaxis paper are available in Microsoft XL. All data requests should be addressed to Professor F. Chappuis: Francois.Chappuis@hcuge.ch.
